# Multimodal imaging features of a spontaneously resolved unilateral congenital macrovessel-related macular edema in a 13-year-old boy

**DOI:** 10.3205/oc000167

**Published:** 2020-08-28

**Authors:** Sefik Can Ipek, Salih Kavukcu, Süleyman Men, Ali Osman Saatci

**Affiliations:** 1 Department of Ophthalmology, Dokuz Eylul University, Izmir, Turkey; 2 Division of Pediatric Nephrology, Department of Pediatrics, Dokuz Eylul University, Izmir, Turkey; 3 Department of Radiology, Dokuz Eylul University, Izmir, Turkey

**Keywords:** congenital retinal macrovessel, retinal arteriovenous malformation, fluorescein angiography, OCT, OCTA

## Abstract

**Purpose:** Congenital retinal macrovessels (CRM) are large aberrant blood vessels that cross the horizontal raphe. Visual acuity may be negatively affected in cases afflicted with CRM due to macular serous detachment, hemorrhage, exudation, foveolar cyst formation and retinal artery occlusion. Even the presence of an anomalous vessel itself running through the foveal avascular zone may compromise the visual acuity. In this case report, we discuss the multimodal imaging characteristics of a case of symptomatic unilateral CRM-related cystoid macular edema and serous macular detachment in a 13-year-old boy.

**Observations:** Optical coherence tomography angiography (OCTA) imaging of the superficial retina revealed the aberrant vessel and anomalous arteriovenous communication between the aberrant vessel and the retinal artery. The foveal avascular zone also appeared partly distorted.

**Conclusions:** The present case reports the second-youngest patient afflicted with unilateral CRM and associated macular edema. There was spontaneous resolution of macular edema within six weeks. In light of the observations in this case and the literature review, the need to refrain from rapid therapeutic intervention in the eyes of patients affected with CRM and macular edema must be emphasized.

## Introduction

Congenital retinal macrovessel (CRM) is a term coined by Brown et al. [1], implying a large aberrant blood vessel (an artery alone, vein alone, or an artery and a vein together) that crosses the horizontal raphe. The macrovessel usually has large tributaries extending on either side of the horizontal raphe. They are believed to originate from abnormal embryological development during gestation. The condition seems to be unilateral, relatively benign in nature, and ophthalmoscopically stable. However, when CRM are associated with macular hemorrhage [2], foveolar cyst formation, serous macular detachment [3], retinal artery occlusion, or the presence of an anomalous vessel running through the foveal avascular zone [4], they may negatively affect vision.

We hereby highlight the multimodal imaging features of a case of unilateral macular macrovessel associated with cystoid macular edema and serous macular detachment in a 13-year-old boy that resolved spontaneously.

## Case description

**Figure 1:**
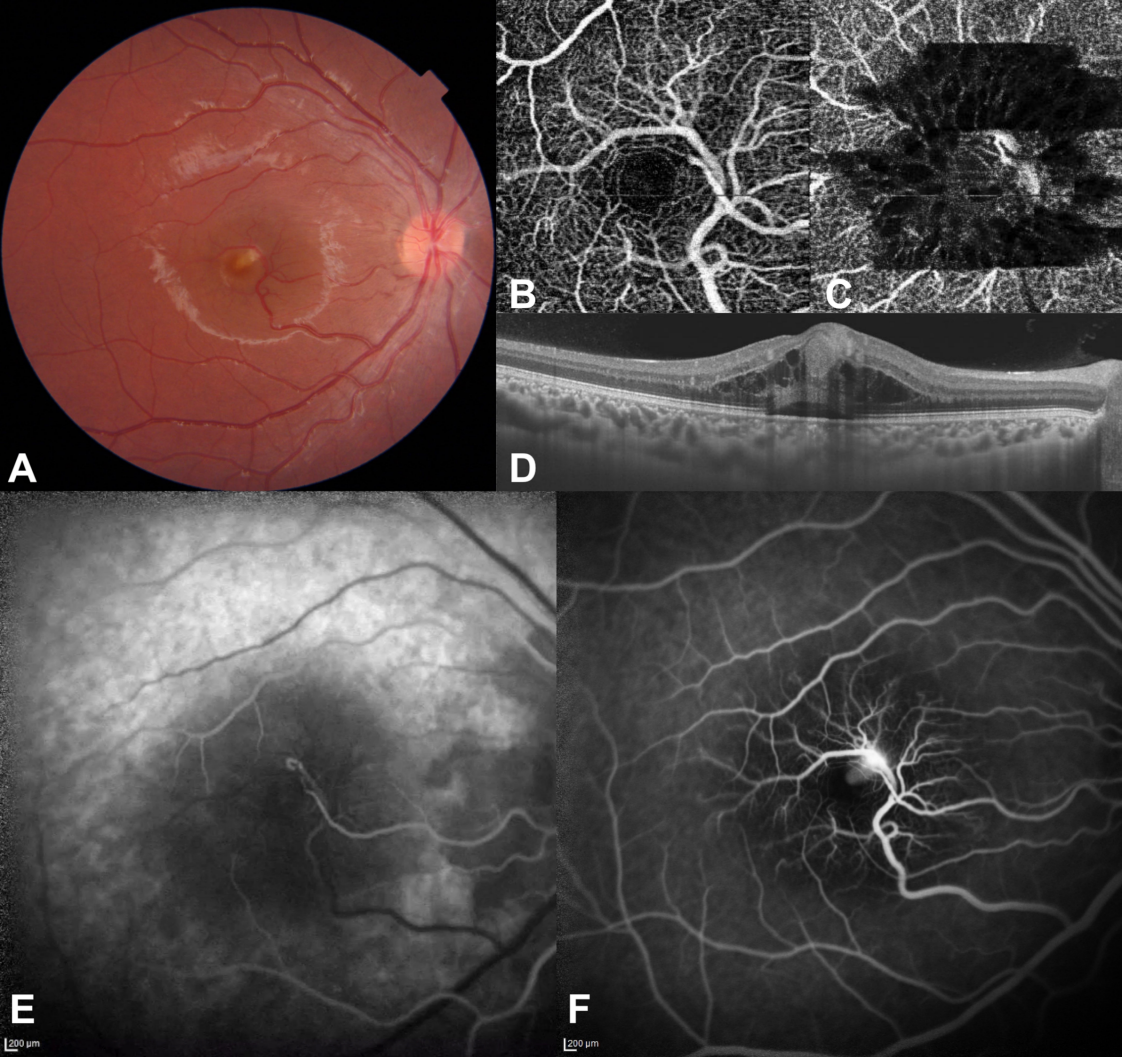
Image findings at initial visit, right eye A) Fundus photograph demonstrating an aberrant retinal venous macrovessel draining into the inferotemporal major vascular arcade vein. A suspected anomalous arteriovenous communication with retinal artery can be detected. A yellowish white area is also seen on the fovea around the arteriovenous communication. B) An OCTA image of the superficial capillary plexus slab demonstrating an aberrant vessel, an anomalous arteriovenous communication between the aberrant vessel and the retinal artery, and a distorted foveal avascular zone. C) An OCTA image of the deep capillary plexus slab showing shadowing and projection artefacts due to the aberrant vessel. Cystoid macular edema can also be seen. D) OCT image depicting cystoid macular edema and serous macular detachment. A few hyperreflective dots in the ganglion nerve fiber layer with backshadowing can also be seen along the course of the aberrant vessel around the fovea. E) Fluorescein angiogram (arterial phase) demonstrating early filling of the venous macrovessel with anomalous arteriovenous communication. Watershed zone of the choroid around the optic disc can also be seen. F) Fluorescein angiogram (late venous phase) demonstrating late dye leakage and pooling from the anastomotic vessel.

A 13-year-old otherwise healthy boy with a history of visual decline in his right eye of one-week duration was examined. He had an unremarkable past medical history and no history of ocular trauma or ocular surgery. On examination, best-corrected visual acuity (BCVA) was 20/100 in the right eye and 20/20 in the left eye. Anterior segment of either eye was unremarkable. Fundus examination of the right eye revealed a normal optic disc with an aberrant retinal venous macrovessel crossing the horizontal raphe and draining into the inferotemporal major vascular arcade vein. There appeared to be a suspicious anomalous arteriovenous communication with a retinal artery (Figure 1A (Fig. 1)). A foveal yellow-whitish spot was also observed, most likely representing the site of arteriovenous communication and the possible site of leakage. The left fundus was normal on examination.

Optical coherence tomography angiography (OCTA) imaging of the superficial vascular plexus in the right fundus revealed the presence of an aberrant vessel, an anomalous arteriovenous communication of the aberrant vessel with the retinal artery, and a distorted foveal avascular zone (Figure 1B (Fig. 1)). The deep vascular plexus slab of the right fundus revealed shadowing and projection artefacts due to the aberrant vessel and cystoid macular edema (Figure 1C (Fig. 1)). The B-scan OCT image showed cystoid macular edema and serous macular detachment. A few hyperreflective dots in the ganglion nerve fiber layer with backshadowing were also seen along the course of the aberrant vessel around the fovea (Figure 1D (Fig. 1)). Fluorescein angiography (FA) showed early filling and delayed emptying of the venous macrovessel with anomalous arteriovenous communications. The macrovessel had numerous branches spreading in the macula. Watershed zone of the choroid around the optic disc was also noticed in the early phase of the angiogram (Figure 1E (Fig. 1)). Capillary bed abnormalities, late dye leakage and pooling from the anastomotic vessel were also noted (Figure 1F (Fig. 1)). A video clip featuring the early fluorescein passage was also obtained (Attachment 1). A magnetic resonance imaging (MRI) of the brain was obtained to rule out any associated intracranial venous malformation, which however did not reveal any abnormalities. The patient was advised to follow up and no treatment was initiated.

**Figure 2:**
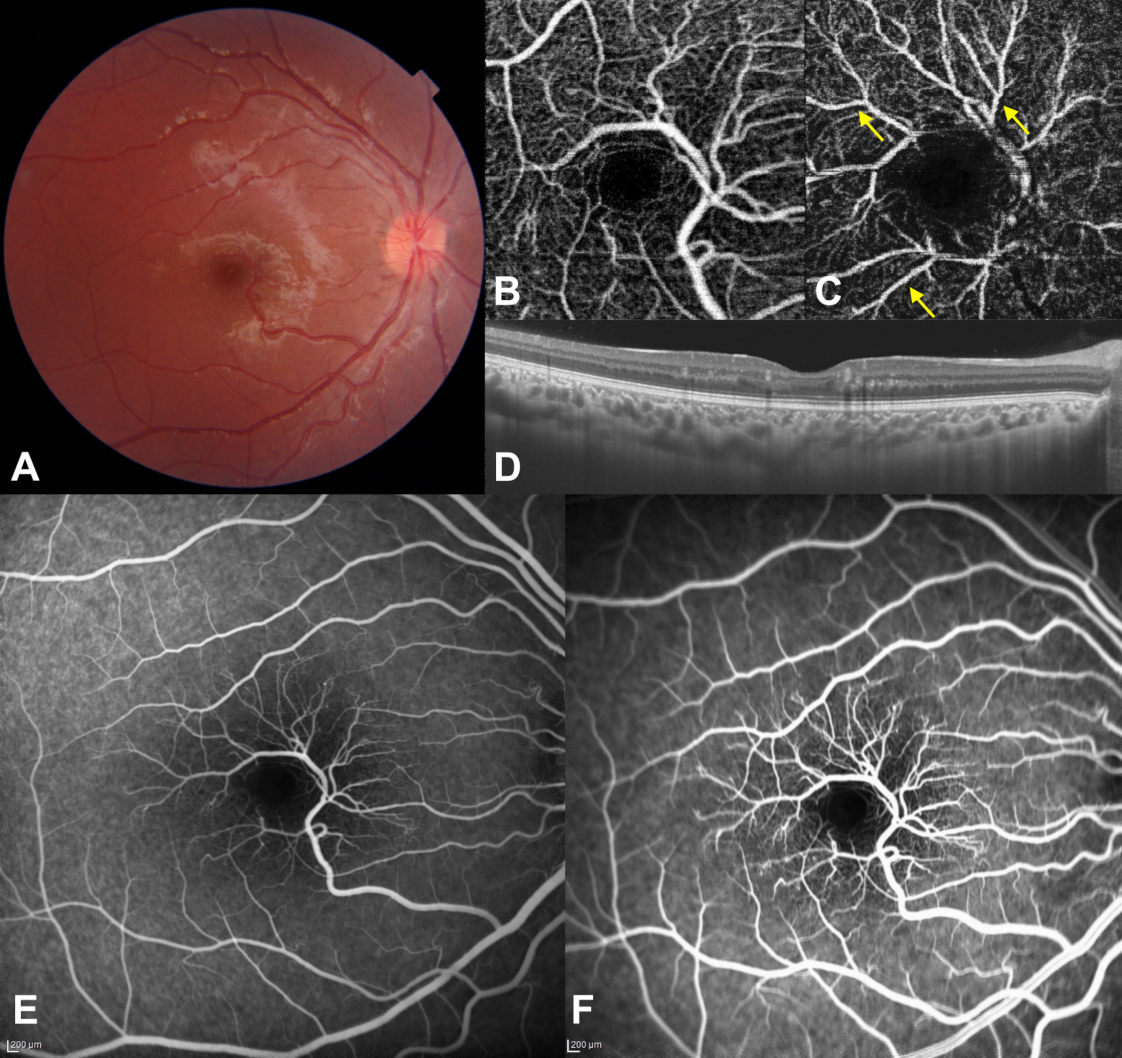
Image findings at six weeks, right eye A) Fundus photograph demonstrating regression of the macular yellowish white lesion. Stellate, fine, hard exudates forming an incomplete macular fan can also be noticed. B) An OCTA image of the superficial capillary plexus slab demonstrating an aberrant vessel and an anomalous arteriovenous communication. A decreased vascular density can be seen around the foveal avascular zone. C) An OCTA image of the deep capillary plexus slab demonstrating a regression of shadowing and projection due to cystoid macular edema and serous macular detachment. Branches of the macrovessel can be identified clearly (arrows). D) OCT image depicting complete regression of cystoid macular edema and serous macular detachment with a normal foveal contour. Multiple hyperreflective dots can be seen at the ganglion nerve fiber and outer nuclear layers. E) Fluorescein angiogram (early venous phase) demonstrating abnormal vascular branches without leakage. F) Fluorescein angiogram (late venous phase) demonstrating abnormal vascular branches and microvascular abnormalities without leakage or pooling of the dye.

At a follow-up of six weeks, the patient’s right eye recorded a BCVA of 20/32. Fundus examination of the right eye revealed the formation of a few stellate, fine, hard exudates, with a significant regression of the yellowish white lesion seen during the patient’s earlier visit (Figure 2A (Fig. 2)). OCTA imaging of the right eye showed abnormal vascular anastomoses with the retinal macrovessels in the superficial vascular plexus slab (Figure 2B (Fig. 2)) and microvascular changes in the deep vascular plexus slab. Branches of the macrovessel could be detected more clearly on the deep capillary plexus slab (Figure 2C (Fig. 2)). A reduced vascular density was seen around the foveal avascular zone. However, the cystoid macular edema and serous macular detachment detected during the earlier OCT imaging were not to be seen (Figure 2D (Fig. 2)). FA imaging showed no leakage or pooling (Figure 2E, F (Fig. 2)).

## Discussion

CRM are most often detected in a routine eye examination and are typically associated with a normal visual acuity. The largest study on CRM was conducted by Pichi et al [5]. They reviewed the records of 49 patients with unilateral CRM in a cross-sectional, retrospective, and multicentric study. In all the reported cases, the CRM had a tortuous, dilated vein. Thirty-nine patients (80%) did not have any evidence of ophthalmic complications. An incidental diagnosis of CRM was made in ten patients (20%) who presented with retinal complications. Among all patients diagnosed with this condition, two eyes had serous macular detachment, and two showed evidence of intraretinal hemorrhage. However, none of the aforementioned eyes received any treatment. An interesting observation was that the youngest patient was 18 years of age. Dramatically, 12 of the 49 patients (24%) in the study had a venous malformation of the brain as detected on an MRI.

The FA findings of CRM can be summarized as: early filling and delayed emptying of the macro vessel, evidence of anastomoses between the major retinal vessel and the aberrant vessel, occasional microvascular capillary bed anomalies, and less frequent dye leakage from the microvascular bed, or a blocked fluorescence from the retinal hemorrhage [1].

In yet another study, OCTA was performed for 17 eyes diagnosed of CRM in 49 patients (35%). The diagnosed cases displayed an increase in vascular flow compared to the surrounding normal veins [5]. The clearly identifiable macro vessels in the superficial capillary plexus slab in 13 eyes illustrated microvascular capillary abnormalities surrounding them. However, these microvascular abnormalities were more evident in the deep capillary plexus compared to the superficial capillary plexus slab. Sebrow et al. [6] described two patients with CRM associated with macroaneurysms. OCTA showed a reduction in the size of the foveal avascular zone in both afflicted eyes owing to a network of capillaries within the central macula. Preziosa et al. [7] described CRM associated with cystoid macular edema in a 25-year-old healthy woman. With the help of OCTA, the macrovessel was localized in the capillary plexus slab and the microvascular abnormalities were predominantly visualized in the intermediate and deep capillary plexus. At one week follow-up, the OCT displayed spontaneous resolution of the edema, the condition remained stable at the immediate monthly follow-up.

Chawla et al. [8] reported a case of a 12-year-old boy who presented with unilateral low visual acuity since childhood. An aberrant retinal macro vessel was observed which had the likelihood of being a vein. OCT imaging showed hyperreflective dots in the outer plexiform and inner nuclear layers, possibly representing hard exudates. OCTA imaging of the superficial retina clearly revealed the abnormal vessels, and a distorted and smaller foveal avascular zone. En face imaging of the deep layers also revealed the abnormal vessels owing to shadowing and projection artefacts.

A brain MRI was also performed in order to rule out any cerebral vascular abnormality as pointed out by Pichi et al [5]. Of 49 patients with CRM, the brain MRI of 12 patients showed evidence of neurovascular lesions. They proposed that an MRI study of the brain should be indicated for every case of CRM.

## Conclusions

Macular edema related to CRM is generally a benign entity, and resolution of edema may occur spontaneously. This case of unilateral CRM with macular edema is that of the second-youngest patient reported in the literature. OCTA can yield additional information in cases of CRM. In the light of the observations of this case and the literature review, the need to refrain from a rapid therapeutic intervention in the eyes of patients with CRM, and macular edema must be emphasized, underscoring the importance of thorough investigations.

## Notes

### Competing interests

The authors declare that they have no competing interests.

### Informed consent

Informed consent has been obtained from the patient’s legal guardian for the publication of this case report.

## Supplementary Material

Video: Right eye, early fluorescein angiogram at the initial presentation
